# Evolution of resistance to MAPK-targeted therapies

**DOI:** 10.1186/1479-5876-13-S1-K2

**Published:** 2015-01-15

**Authors:** Roger Lo

**Affiliations:** 1 Melanoma Clinic in Dermatology Member, Jonsson Comprehensive Cancer Center David Geffen School of Medicine at UCLA, Los Angeles, California, USA

## 

BRAF inhibitors (BRAFi) elicit rapid antitumor responses in the majority of patients with ^V600^BRAF mutant melanoma, but acquired resistance is almost universal. Early understanding of how melanomas acquire resistance to BRAFi via MAPK pathway reactivation has guided the development of specific BRAFi-anchored inhibitor combinations designed to overcome resistance. The first of these successful combinations has been between BRAF and MEK inhibitors. Combined BRAF/MEK targeted therapy improves upon BRAFi therapy but is still beset by acquired resistance. Thus, studying how melanomas escape from BRAFi and how these processes are similar or distinct from acquired resistance mechanisms to BRAFi+MEKi remains of the utmost importance to melanoma therapeutics. Recent whole-exome analysis of patient-paired melanoma samples obtained pre-BRAFi treatment and post-disease progression after initial responses have provided landscape genetic perspectives into the nature of tumor heterogeneity, clonal evolution, and core resistance pathways. This benchmark understanding is helping to guide and prioritize clinical studies of BRAFi-based combinations such as that between BRAFi and AKTi. Recent work on the genetic mechanisms of acquired BRAFi+MEKi resistance has shed key insights into the molecular limitations of this therapeutic approach but also novel therapeutic opportunities. Overall, genetic alterations affecting individual genes are not highly recurrent and collectively cannot account for a significant subset of clinical acquired resistance to BRAF or combined BRAF/MEK targeted therapies (MAPKi). Thus, understanding the entire spectrum of genetic and non-genetic mechanisms of acquired MAPKi resistance and the temporal continuum of these evolutionary processes promises to usher in a new era of personalized medicine for melanoma patients.

